# Mechanistic Insights into Membrane Protein Clustering Revealed by Visualizing EGFR Secretion

**DOI:** 10.34133/2022/9835035

**Published:** 2022-10-16

**Authors:** Haijiao Xu, Jinrui Zhang, Yijia Zhou, Guanfang Zhao, Mingjun Cai, Jing Gao, Lina Shao, Yan Shi, Hongru Li, Hongbin Ji, Yikai Zhao, Hongda Wang

**Affiliations:** ^1^State Key Laboratory of Electroanalytical Chemistry, Changchun Institute of Applied Chemistry, Chinese Academy of Sciences, Changchun, 130022 Jilin, China; ^2^State Key Laboratory of Cell Biology, Shanghai Institute of Biochemistry and Cell Biology, Center for Excellence in Molecular Cell Science, Chinese Academy of Sciences, Shanghai 200031, China; ^3^School of Life Science and Technology, ShanghaiTech University, Shanghai 200031, China; ^4^University of Science and Technology of China, Hefei, 230026 Anhui, China; ^5^University of Chinese Academy of Sciences, Beijing 100049, China; ^6^School of Life Science, Hangzhou Institute for Advanced Study, University of Chinese Academy of Sciences, Hangzhou 130102, China; ^7^Laboratory for Marine Biology and Biotechnology, Qingdao National Laboratory for Marine Science and Technology, Qingdao, 266237 Shandong, China

## Abstract

Most plasmalemmal proteins are organized into clusters to modulate various cellular functions. However, the machineries that regulate protein clustering remain largely unclear. Here, with EGFR as an example, we directly and in detail visualized the entire process of EGFR from synthesis to secretion onto the plasma membrane (PM) using a high-speed, high-resolution spinning-disk confocal microscope. First, colocalization imaging revealed that EGFR secretory vesicles underwent transport from the ER to the Golgi to the PM, eventually forming different distribution forms on the apical and basal membranes; that is, most EGFR formed larger clusters on the apical membrane than the basal membrane. A dynamic tracking image and further siRNA interference experiment confirmed that fusion of secretory vesicles with the plasma membrane led to EGFR clusters, and we showed that EGFR PM clustering may be intimately related to EGFR signaling and cell proliferation. Finally, we found that the size and origin of the secretory vesicles themselves may determine the difference in the distribution patterns of EGFR on the PM. More importantly, we showed that actin influenced the EGFR distribution by controlling the fusion of secretory vesicles with the PM. Collectively, a comprehensive understanding of the EGFR secretion process helps us to unravel the EGFR clustering process and elucidate the key factors determining the differences in the spatial distribution of EGFR PM, highlighting the correlation between EGFR secretion and its PM distribution pattern.

## 1. Introduction

Membrane proteins, as essential components of biological membranes, are involved in diverse cellular processes, such as cell adhesion, cell signaling, accumulation, and energy transfer [1]. It is well established that an array of membrane proteins are organized into clusters to perform their cellular functions. For instance, we have utilized superresolution imaging to reveal that multiple proteins, such as GLUT1, EGFR, EpCAM, and P-glycoprotein, exist mainly as nanosized clusters in the plasma membrane (PM) [2–5]. The nanometer-scale clustering of membrane proteins facilitates intercommunication and thus improves signaling efficiency and information processing [6]. A thorough knowledge of the clustering mechanisms of membrane proteins is required to gain insight into the relationships between their dynamic organization and physiological functions. Several previous studies have focused on the physical principles and mechanisms underlying functional membrane protein clustering, some of which demonstrated that protein–protein interactions are necessary to induce clustering, and others believed that lipid rafts may also regulate cluster formation [7, 8]. Nevertheless, the mechanisms of the cluster formation are still incompletely understood, and in particular, direct evidence on the process and mechanistic details of the clustering is lacking.

The epidermal growth factor receptor (EGFR) is a well-characterized cluster-based receptor tyrosine kinase that initiates signaling cascades promoting cell growth, proliferation, and differentiation and is therefore implicated in a variety of human cancers [9–12]. Notably, in previous work [3, 13], we have gained mechanistic insight into EGFR clustering by superresolution imaging, revealing that EGFR clusters existed in both activated and resting cells and that they differed on the apical and basal surfaces, i.e., clusters were more and larger on the apical membrane than on the basal membrane. In addition, we have provided direct evidence for the involvement of lipid rafts in cluster formation. Going further, we aimed to uncover the process and mechanistic details of EGFR clustering and elucidate key factors determining the differences in EGFR clusters on the apical and basal cell surfaces.

The life of EGFR begins at the endoplasmic reticulum (ER), where they are synthesized, folded, and assembled and then transported to the Golgi complex for processing and maturation [14]. Mature receptors are subsequently delivered to the PM via the trans-Golgi network (TGN), the major protein sorting station [15]. Within the secretory pathway, membrane trafficking is responsible for the transport of membrane proteins from their site of synthesis in the ER via the Golgi apparatus to their final site of action on the cell surface, mainly involving COPII vesicle-regulated ER export to the Golgi complex and post-Golgi vesicle-mediated transport from the TGN to the PM [16–20]. Compared with extensive work on characterizing the EGFR endocytic pathway [21, 22], little is known about its secretion pathway in detail, especially the newly synthesized EGFRs. Direct visualization of the entire process of EGFR de novo secretion will facilitate the elucidation of the EGFR clustering process and hence contribute to the elaboration of the mechanistic details.

The last but most important stage of secretion is fusion of secretory vesicles with the PM. A prior report established that GLUT4 secretion onto the PM involves two methods: fusion with release and fusion with retention [23]. The former leads to GLUT4 clustering, while the latter causes GLUT4 molecules to disperse on the PM. In this light, we wondered whether the pattern of EGFR distribution on the apical and basal membranes varied depending on these two fusion methods. If so, we need to elucidate the primary factors that determine which type of fusion method is utilized by EGFR-secreting vesicles. Overwhelming evidence has shown that this cytoskeletal actin is closely intertwined with the fusion of secretory vesicles with the PM [24]. Moreover, there are data suggesting that cortical actin participates in EGFR cluster formation [25]. However, the mechanism by which actin regulates the PM distribution of EGFR is still not deeply understood. Thus, it is of great significance to examine whether actin determines the distribution form of EGFR on the PM by controlling the fusion method of secretory vesicles with the PM.

In this study, a spinning–disk confocal microscope with high spatiotemporal resolution, which we previously used to reveal the structural mechanism of orderly and efficient vesicle transport during EGFR endocytosis [26], was employed to elucidate the correlation between EGFR secretion and EGFR PM clusters in resting cells. With the aim, we localized their sites of exocytosis, analyzed their distribution patterns on the apical and basal plasma membranes, and tracked the clustering process. Furthermore, we probed the main cause of EGFR distribution pattern differences on the apical and basal membranes, suggesting that secretory vesicles themselves and the actin-associated fusion method with the PM determine such distinctness. This work presents a novel idea for elucidating the mechanisms of EGFR clustering by taking its origin as the entry point.

## 2. Results and Discussion

### 2.1. Visualization of the Intracellular EGFR Distribution Profile and Secretion Route

**Figure 1:**
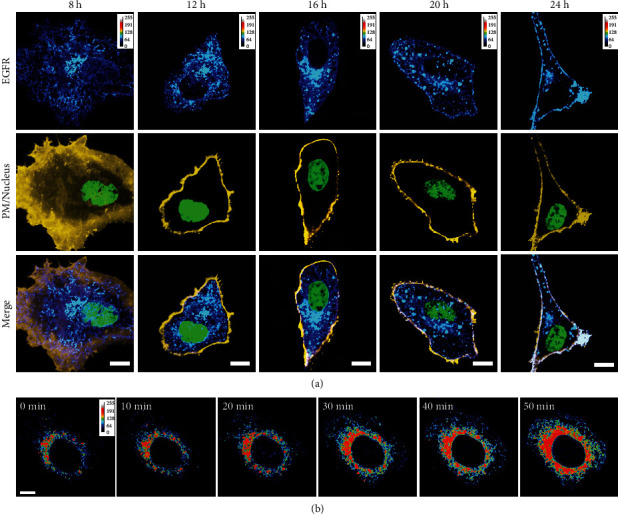
Direct visualization of the biosynthetic trafficking route of EGFR. (a) EGFR is localized to multiple sites as secretion progresses. The A549 cells expressing GFP-EGFR for the indicated times were fixed and then incubated with DiD (yellow) and DAPI (green) to label the cell membrane and nucleus, respectively. (b) Selective frames from time serial imaging show the process of EGFR synthesis from less to progressively more in the perinuclear region. The A549 cells were transiently transfected with GFP-EGFR. The 0 min time point represents EGFR expression for 4 h. Scale bars = 10 *μ*m.

To observe the subcellular distribution and localization of newly synthesized EGFR, we first transiently transfected A549 cells with GFP-EGFR and labelled the PM and nucleus at different time points of GFP-EGFR expression. To ensure the integrity of EGFR functionality, we utilized Western blot analysis to confirm the successful expression of full-length GFP-EGFR (Figure S1). Subsequently, we performed three-color confocal imaging analysis on these cells (Figure 1(a)). At 8 h after transfection, GFP-EGFR resided in internal structures most likely resembling ER. The ER is functionally divided into two types, the rough ER, which is located near the nuclear envelope (NE) and responsible for membrane protein biosynthesis, and smooth ER, which is tubular in charge of the assembly of the COPII vesicles mediating ER-to-Golgi traffic [27, 28]. Accordingly, this observation suggested that at this time point, EGFR was synthesized primarily in the rough ER, and some of the newly synthesized EGFR was distributed to the smooth ER ready to export. Notably, our real-time imaging results obtained by spinning-disk confocal microscopy demonstrated a progression of EGFR synthesis from small to large amounts in the rough ER proximal nuclear region, with the appearance of tubular ER-like structures throughout the cell (Figure 1(b)). After 12 h of expression, GFP-EGFR localized mostly to the ER reticular region and, to a much lesser extent, to vesicle-like structures. At 16 h of expression, EGFR was significantly depleted from the ER-like structure, most of which was localized to the Golgi complex-like structure, and some was located in vesicles distributed in the cytoplasm. As the expression time increased (20 h expression), the EGFR-containing vesicles increased strongly, and the colocalization image of GFP-EGFR with the PM indicated that a small part was already close to the PM and anchored on it. At 24 h of expression, we observed that the majority of EGFR was apparently distributed on the plasma membrane, as illustrated by the intense overlapping signals of EGFR and PM.

The above data suggested that nascently biosynthesized EGFR passed through multiple positions for delivery to the cell surface. To preclude the possibility that the above observed EGFR behavior is caused by the coupled GFP, we used GFP alone as a control and monitored its synthesis-sorting process (Figure S2). Our observations showed that GFP was diffusely distributed throughout the cell in the whole secretion process, thus suggesting that the above secretory pathway was experienced by EGFR itself. Simultaneously, we also examined the distribution profile of constitutively activated mutant of EGFR (EGFR-L858R mutant) in the secretion process. Consistently, EGFR-L858R also underwent the same route of secretion as EGFR wild-type (Figure S3).

**Figure 2:**
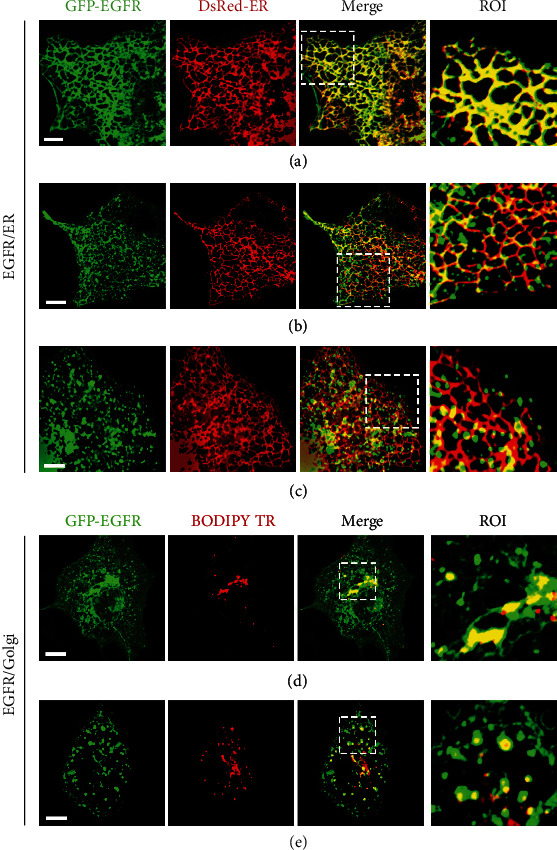
Association analysis of EGFR with ER/Golgi in the secretory pathway. (a–c) Two-color colocalization images of EGFR with ER in different stages of secretion. COS-7 cells were cotransfected with GFP-EGFR and DsRed-ER. (d, e) Colocalization levels of EGFR with the Golgi apparatus in different stages of secretion. The A549 cells were transfected with GFP-EGFR and labelled with BODIPY TR. Scale bars = 10 *μ*m.

To precisely verify the involvement of ER in EGFR secretion, we transiently cotransfected COS-7 cells with GFP-EGFR and DsRed-ER and then analyzed their relationship using two-color fluorescence colocalization imaging.  Figure 2(a) shows that the tubular structure located by GFP-EGFR completely overlapped with the ER reticular region, indicating that the ER is indeed the origin site of EGFR life. In another association between EGFR and ER, we found that EGFR-containing vesicles were formed (Figure 2(b)), as shown in the magnified image of the region of interest (ROI). We assumed that these vesicles may result from the ER export of EGFR. Moreover, in an alternative colocalization image of EGFR with the ER (Figure S4), EGFR displayed a marked punctate distribution at three-way junctions of the ER. The three-way junctions are considered as key elements of the ER network and form when the tip of an ER tubule fuses to the side of another tubule [27]. Here, we surmised that the three-way junctions may act as budding sites of EGFR-secreting vesicles. In Figure 2(c), the magnified ROI image exhibited another distinct phenomenon in which EGFR was almost exported from the ER tubule. These results provide a clear and comprehensive picture of the different stages of the interrelationship between EGFR and ER.

In addition, we conducted colocalization analysis of newly synthesized EGFR with BODIPY TR, a well-known marker for the Golgi apparatus and Golgi-associated vesicles [29], to directly reveal their correlation. As depicted in Figure 2(d), EGFR showed a fairly strong colocalization with the Golgi cisternae. This result indicated that EGFR was transported to the Golgi apparatus for further processing. Furthermore, in Figure 2(e), we observed that EGFR evidently colocalized with Golgi-associated vesicles, implying that post-Golgi vesicles may participate in EGFR transport to the plasma membrane. The importance of the Golgi for EGFR transport onto the cell membrane was further determined using BFA, which is known to inhibit both transport from the ER to the Golgi and from the Golgi to the plasma membrane [30]. To exclusively impede transport from the Golgi to the plasma membrane, the inhibitory effect of BFA on ER-to-Golgi transport needed to be excluded; thus, we assayed the fate of EGFR that already localized to Golgi in the presence or absence of BFA treatment (Figure S5). As shown in Figure S5A, at 10 h of expression, EGFR was primarily concentrated in the Golgi region. In the control group, the cells were chased for 20 h without BFA treatment, showing that a large number of EGFR signals appeared on the plasma membrane (Figure S5B). In contrast, when cells were subjected to continued incubation for 20 h under maintaining BFA treatment conditions, we observed a substantial accumulation of EGFR in the Golgi complex and the return of some EGFR to the ER (Figure S5C). These data indicated that the Golgi apparatus played a decisive role in EGFR secretion onto the plasma membrane.

### 2.2. Uncovering the Mechanism of EGFR PM Clustering and Its Associated Functions

**Figure 3:**
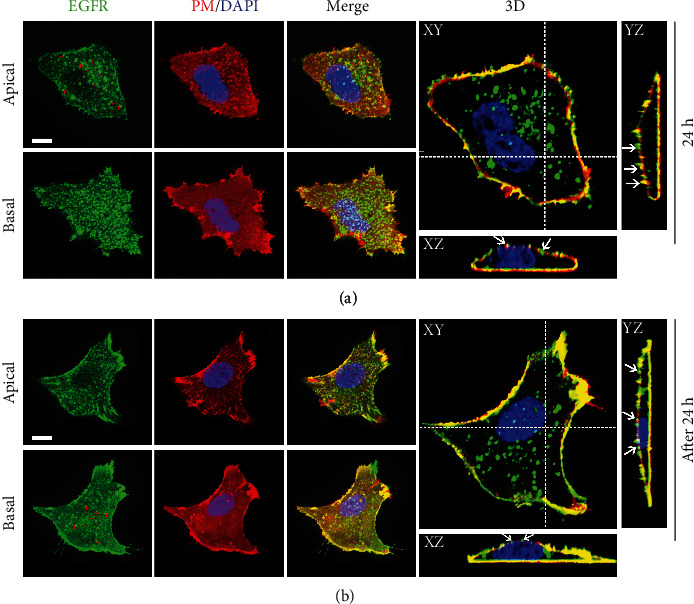
Spatial distribution pattern of EGFR in the PM of resting cells. A549 cells expressing GFP-EGFR for 24 h (a) or beyond 24 h (b) were stained with DiD and DAPI. Individual cells were imaged using three-color 3D confocal microscopy. The upper panels show the representative cells on the apical state. The lower panels show the representative cells on the basal state. Red crosses mark the anchored EGFR secretory vesicles. Serial confocal sections were collected from the top to the bottom of the representative cell (right panels in (a) and (b)). The dashed lines show the positions from which the *XZ* and *YZ* sections were taken. Arrowheads mark the anchored EGFR secretory vesicles (a) and the EGFR clusters (b). Bars, 10 *μ*m.

We have previously shown that EGFR exists as clusters on the PM with distinct forms on the apical and basal membranes [3]. To further address the distribution patterns of EGFR on the apical and basal membranes in resting cells, we executed three-color three-dimensional (3D) confocal imaging on cells expressing GFP-EGFR 24 h and after 24 h followed by labelling the cell membrane and nucleus. At 24 h of EGFR expression (Figure 3(a)), we observed numerous EGFR-containing vesicles anchored to the apical membrane, as marked by the red crosses on the apical membrane. The spot-like structures indicated by white arrows in the *XZ* and *YZ* sections also represent anchored vesicles. More visibly, we found that many EGFR vesicles anchored to the basal membrane. Furthermore, the dynamic imaging results showed that the movement of these vesicles near the apical membrane (Movie S1) and basal membrane (Movie S2) was restricted and almost stationary. Therefore, we speculated that these anchored vesicles may act as secretory “hot spots” waiting to fuse with the plasma membrane, as previously described in polarized cells [31]. Our data suggested that a mechanism for the restricted delivery was in place in nonpolarized cells as well.

Expression for more than 24 h can ensure that the majority of EGFR has been uploaded to the plasma membrane. Figure 3(b) shows the distribution patterns of EGFR on the apical and basal cell membranes. On the apical membrane, compared to the above, anchored large vesicles were significantly reduced, and these spot-like structures indicated by arrows in the *XZ* and *YZ* plane images may represent formed EGFR clusters. Likewise, on the basal membrane, only a few secretory hot spots were observed, as marked by red crosses. Most EGFR was uniformly distributed as relatively smaller clusters on the basal membrane, as demonstrated by the basal image and *XZ* and *YZ* plane images. Together, consistent with our prior results [3], these data indicated that in resting cells, a significant fraction of PM EGFR was organized into distinct clusters, i.e., EGFR formed larger clusters on the apical membrane than the basal membrane. It is worth mentioning that we revealed that EGFR-L858R mutant, which we have already observed to possess the same secretory pathway as EGFR wild-type (Figure S3), also exhibited the identical distribution characteristics as EGFR wild-type on the plasma membrane, i.e., it appeared as a relatively large cluster on the apical membrane (Figure S6). Unfortunately, the detailed mechanism of EGFR clustering, let alone the regulators of differences in clusters, is unclear and deserves to be further explored.

**Figure 4:**
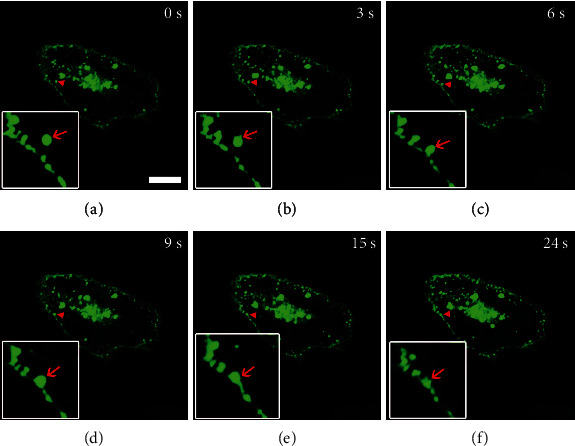
Fusion of EGFR secretory vesicles with the PM. A549 cells were transfected with EGFR for 16 h and then tracked by real-time imaging using a confocal microscope. Images in (a–f) are selected images from time-lapse sequences. The zoomed-in time-lapse images clearly illustrate the dynamic process of a single EGFR-secreting vesicle fusing with the plasma membrane and forming a cluster. Bar, 10 *μ*m.

Since PM EGFR is derived from the secretion pathway, we intended to investigate the relationship between EGFR exocytosis and its PM clusters. For this purpose, we monitored the fusion process of a single secretory vesicle on the cell expressing GFP-EGFR for 20 h.  Figure 4(a) shows that EGFR was mostly localized in the perinuclear Golgi-like region and that some EGFR has arrived at the cell membrane. Furthermore, as indicated by red arrows, time-lapse confocal recordings clearly illustrated a process, whereby a single EGFR-containing vesicle in the vicinity of the PM gradually moved towards the PM until contact (Figures 4(a)–4(c)) and eventually fused with the PM through sustained interaction (Figures 4(d) and 4(e)). As a result, at this fusion site, the vesicle transformed into a slightly diffused punctate structure (Figure 4(f)), which is an indicator of the formation of an EGFR cluster. Taken together, these real-time imaging results suggested that the EGFR secretory vesicle may be a precursor of the EGFR PM cluster, specifically a secretory vesicle fused to the plasma membrane to form an EGFR cluster.

**Figure 5:**
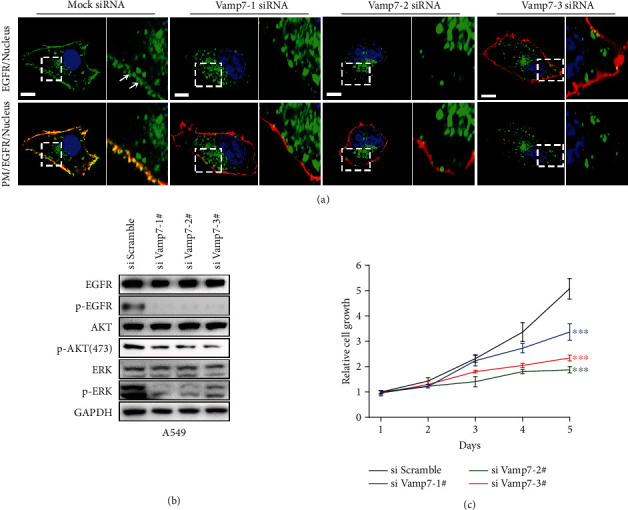
VAMP-7-determined EGFR PM localization and clustering affect EGFR signaling and cell proliferation. (a) Detection of EGFR PM localization and clustering in confocal sections. A549 cells were pretransfected with mock siRNA or three VAMP-7 siRNAs and then transfected with GFP-EGFR (green) finally labeled the cell membrane (red) and nucleus (blue) with Did and DAPI, respectively. White arrows point to EGFR PM clusters. Bars, 10 *μ*m. (b) Western blot analysis of the effect of VAMP-7 knockdown on EGFR activation and its downstream signal transduction in A549 cells. (c) MTT-based cell proliferation assay of A549 cells in the case of normal VAMP-7 expression and VAMP-7 knockdown. The data are shown as the mean ± SD.

We further wanted to investigate the role of vital proteins that are involved in the transport of secretory vesicles to the plasma membrane in EGFR onto PM and EGFR clustering processing. In this regard, we selected VAMP-7 that has been postulated to be a v-SNARE protein for constitutive secretion, operating mainly in the post-Golgi secretion pathway as well as in secretory vesicles-PM fusion process [32, 33], as the subject of our study. We firstly silenced the expression of VAMP-7 in A549 cells by transfecting siRNA, as supported by apparent decrease in VAMP-7 mRNA levels in Figure S7. Then, we performed three-color colocalization imaging to analyze the effect of VAMP-7 on EGFR PM localization and clustering. As shown in Figure 5(a), compared with the control group (Mock siRNA), VAMP-7 knockdown (KD) led to significant retention of EGFR-secreting vesicles in the cytoplasm without reaching PM to generate clusters. This result reinforced that secretory vesicles transport to and further fusion with the plasma membrane is a necessary prerequisite for EGFR PM clustering. Next, we checked whether the depletion of VAMP-7 could have an effect on EGFR signaling. Western blot data showed that VAMP-7 KD inhibited EGFR phosphorylation and the activation of downstream PI3K-Akt and MAPK pathways, as demonstrated by obvious downregulation of the phosphorylation level of EGFR, AKT, and ERK in Figure 5(b). These results confirmed that depletion of VAMP-7 impaired EGFR activation and downstream signaling. Moreover, we examined the effect of VAMP-7 KD on cell proliferation by performing MTT-based growth assay. The MTT assay results revealed that cell growth was obviously inhibited by VAMP-7 depletion (Figure 5(c)). These data indicated that VAMP-7 knockdown has inhibitory effect on EGFR signaling and cell growth, at least partially, by disrupting EGFR localization and clustering on the plasma membrane. To further validate the importance of VAMP-7 on EGFR signaling pathways and cell growth, we conducted siRNA rescue experiments. It was shown that the remediation was able to noticeably recover EGFR signaling process and cell growth rate (Figure S8). Collectively, these results revealed that VAMP-7 regulated EGFR PM localization and clustering, which in turn is vital for EGFR signaling and cell growth.

### 2.3. The Size and Origin of EGFR-Secreting Vesicles Determine the Distribution Pattern of EGFR on the PM

**Figure 6:**
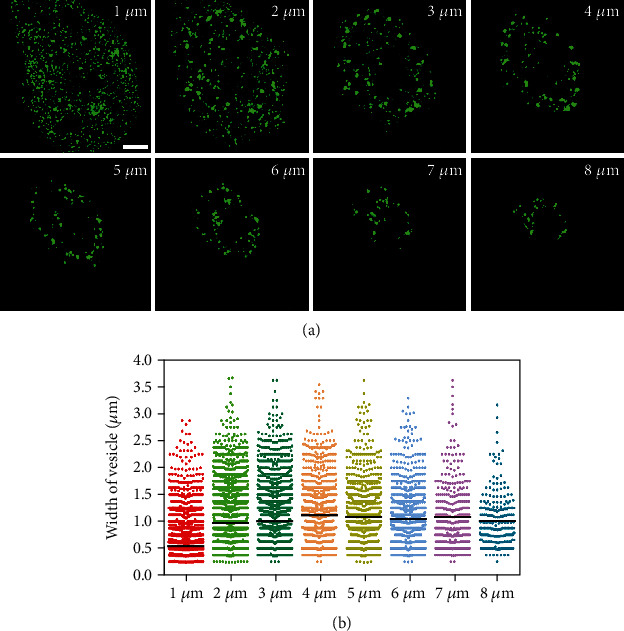
Characterization of EGFR-secreting vesicles in different layers of A549 cells. (a) Sequential *z*-axis planes made at 1, 2, 3, 4, 5, 6, 7, and 8 *μ*m above the basal membrane are shown in cells expressing GFP-EGFR for 16 h. (b) A statistical scatter plot of EGFR-secreting vesicle size distribution at different levels of cells under the same conditions as (a). Each layer of vesicles origin from three different experiments with nearly 100 cells. Bar, 10 *μ*m.

Above, we showed that fusion of the secretory vesicle with the plasma membrane yielded an EGFR cluster; subsequently, we wondered about the causes of the differences in EGFR PM clusters. Based on the above findings, we hypothesized that the size of the secretory vesicles themselves may dictate the size of the EGFR cluster. To test this hypothesis, we first studied the 3D distribution of secretory vesicles by using confocal microscopy recordings at 0.2 *μ*m *z*-axis intervals through A549 cells expressing GFP-EGFR for 16 h. In all experiments, A549 cells grown on glass coverslips reached an average height of approximately 10 *μ*m. Our *z*-axis sections and statistical analysis (Figures 6(a) and 6(b)) indicated that small EGFR-secreting vesicles were mainly concentrated at the proximal base of the cell (1 *μ*m above the basal membrane). In contrast, secretory vesicles of relatively large size were observed between 2 and 8 *μ*m above the basal membrane. We also found that these small secretory vesicles had broader distributions than the larger vesicles, which were excluded from the region near the base. Overall, we reasonably assumed that distinct morphology between apical and basal EGFR secretory vesicles caused the differences in EGFR clusters on these two membranes. Specifically, the larger secretory vesicles on the apical membrane and the smaller secretory vesicles on the basal membrane corresponded to the larger EGFR cluster on the apical membrane and the smaller cluster on the basal membrane, respectively.

**Figure 7:**
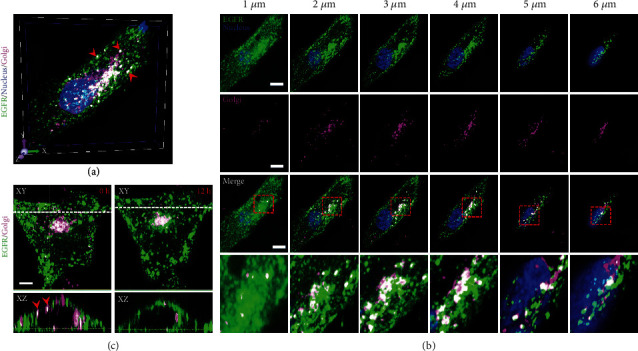
Spatial correlation of EGFR-secreting vesicles with the Golgi apparatus. A549 cells that transfected with GFP-EGFR were labelled with the Golgi marker BODIPY TR and the nuclear dye DAPI. (a) A three-color 3D confocal image of the cell shows strong colocalization of EGFR-secreting vesicles with the Golgi apparatus. Red arrows point to the colocalization of EGFR-secreting vesicles with post-Golgi vesicles. (b) Colocalization analysis of EGFR-secreting vesicles with the Golgi apparatus at 1, 2, 3, 4, 5, and 6 *μ*m above the basal membrane of the cell in (a). (c) EGFR is directly delivered to the apical surface via post-Golgi vesicles. Representative images taken from the time-lapse 3D recordings are shown. Time = 0 h represents EGFR expression for 10 h. Time = 12 h means that EGFR expressed 10 h continued to be tracked for another 12 h. The dashed lines indicate the position of the displayed *XZ* sections. Red arrows point to the EGFR-secreting vesicles that colocalized with Golgi-vesicles. Bars, 10 *μ*m.

Our previous findings established that post-Golgi vesicles are essential for EGFR secretion into the plasma membrane (Figure 2(e) and Figure S5). We next aimed to concretely address the correlation of apical and basal EGFR-secreting vesicles with post-Golgi vesicles by implementing three-color 3D confocal imaging on cells expressing GFP-EGFR with tagging of the nucleus and Golgi apparatus.  Figure 7(a) represents 3D imaging of one cell demonstrating substantial localization of EGFR with the Golgi, including the apparent localization of EGFR secretory vesicles to Golgi-associated vesicles, as indicated by red arrows. Furthermore, sequential *z*-axis recordings revealed more clearly that colocalization of EGFR secretory vesicles with Golgi-associated vesicles occurred mainly 2 *μ*m above the basal membrane up to the apical membrane (Figure 7(b)). These results probably showed direct delivery of apical EGFR-secreting vesicles from the Golgi to the cell surface. To confirm the involvement of post-Golgi transport in EGFR secretion onto the apical membrane, we utilized 3D time-lapse imaging to assay the arrival of EGFR onto the apical membrane. The EGFR and Golgi double-labelled cells were chased for 12 h at 20 min intervals. *XY* and *XZ* reconstructions are shown before and after chasing in Figure 7(c). At time = 0 h, some large EGFR-secreting vesicles colocalized with post-Golgi vesicles and anchored to the apical membrane, as indicated by red arrows in the *XZ* image. After chasing for 12 h, the EGFR signal of the apical surface was brightened integrally, as a result of EGFR reaching the apical membrane, as clearly shown in the *XZ* microscopy section. Thus, these data suggested that post-Golgi vesicles mediated EGFR secretion onto the apical membrane. Notably, some proteins, such as glycosylphosphatidylinositol-anchored proteins (GPI-APs), have been reported to be clustered into lipid rafts in the TGN, and raft association mediates apical transport [34]. Moreover, TGF-*β* receptors have been shown to exist as a cluster in post-Golgi vesicles [35]. Accordingly, we considered that EGFR may be organized into clusters when apical sorting at the TGN, which requires further confirmation in future studies.

### 2.4. Actin Determines EGFR Distribution Patterns on the PM by Governing the Secretory Vesicle–PM Fusion Method

**Figure 8:**
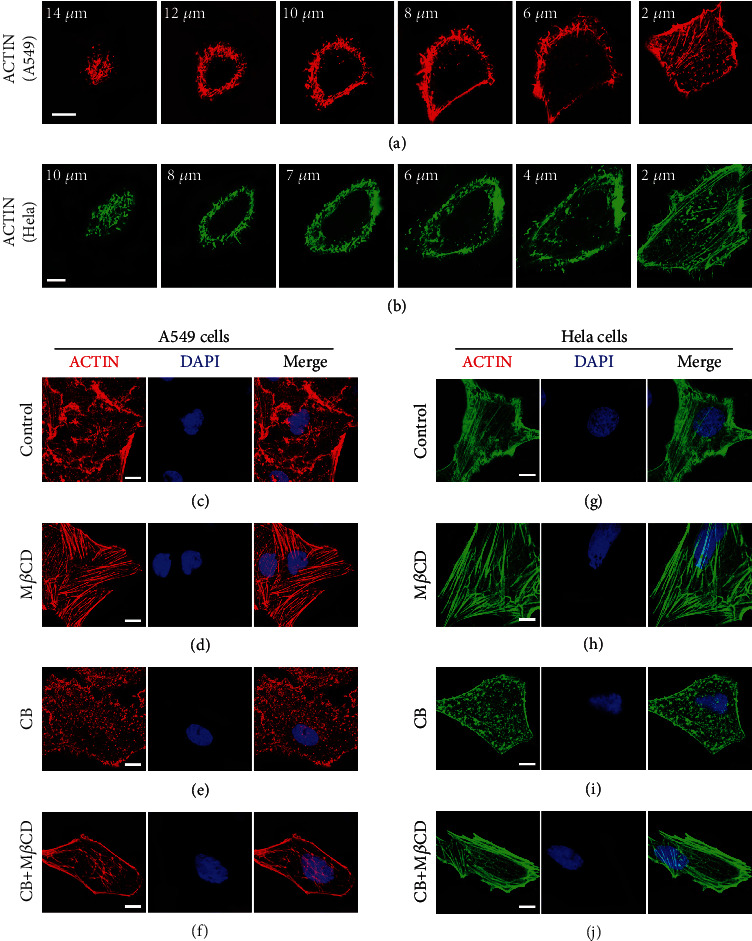
Actin cytoskeleton organization. (a, b) Confocal images of actin organization from representative sections along the apical–basal axis of an A549 cell (a) and a HeLa cell (b). The actin cytoskeleton of cell was labelled with Alexa Fluor 647-conjugated phalloidin. (c–j) Representative images of the actin cytoskeleton from A549 cells or HeLa cells. These cells were untreated (c, g), treated with M*β*CD for 30 min at 37°C (d, h), treated with CB for 30 min at 37°C (e, i) or treated with CB followed by M*β*CD at 37°C (f, j), and fixed and labelled with phalloidin–Alexa Fluor 647 and DAPI (cell nuclei). Each selected image represents the most predominant one of at least 50 different cells from three independent experiments under that experimental condition. Scale bars are all 10 *μ*m.

In view of the involvement of actin in EGFR cluster formation and its regulation of vesicle–PM fusion, we asked whether the actin cortex determined the distribution morphology of EGFR on the PM by controlling the fusion method of secretory vesicles with the PM. The actin cortex of a cell is a thin layer of actin meshwork that uniformly underlies the plasma membrane of the entire cell. To image the actin structure and subcellular distribution in the whole cell under normal physiological conditions, we labelled the actin cytoskeleton of A549 cells with Alexa Fluor 647-conjugated phalloidin and then performed 3D confocal imaging on the cells. We observed that the actin cortex structure and subcellular distribution varied significantly across A549 cells. As shown in Figure 8(a), the actin on the near apical membrane was dense, short, and misaligned, whereas actin on the near basal membrane was sparse and partially existed in the form of aligned actin filaments. By the same token, in HeLa cells, the actin cortex shared a similar structure and distribution pattern with that in A549 cells (Figure 8(b)). Given the importance of the actin cortex in membrane fusion, we surmised that the observed differences in the actin organization forms between the apical and basal membranes may impinge on EGFR secretory vesicle–PM fusion. Thus, this may lead to different forms of EGFR distribution on the cell surface, whereby EGFR was present in larger clusters on the apical membrane but almost dispersed on the basal membrane.

**Figure 9:**
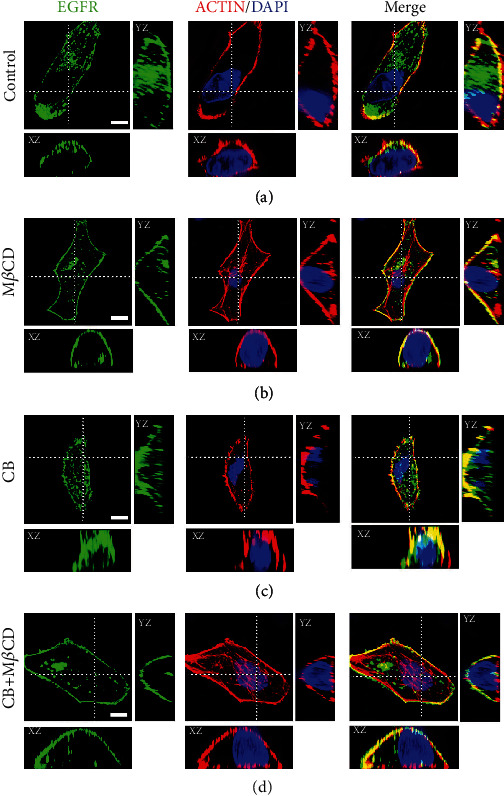
The association of EGFR spatial distribution with actin cytoskeleton morphology. Representative confocal images of A549 cells are *z* sections taken at sequential focal planes. The dashed lines show the positions from which the *XZ* and *YZ* sections were taken. The cells were subjected to the following treatments: (a) control; (b) M*β*CD for 30 min at 37°C; (c) CB alone for 30 min at 37°C; (d) CB for 30 min followed by M*β*CD for another 30 min at 37°C. The cells were fixed simultaneously and processed for actin cytoskeleton labelling using phalloidin–Alexa Fluor 647. Cell nuclei were stained with DAPI. Three independent experiments were performed in triplicate, and at least 50 different cells were observed per group. Scale bars are all 10 *μ*m.

To validate our assumption, we examined the effect of actin structure changes on EGFR secretory vesicle–PM fusion, with the ultimate goal of detecting the resulting changes in EGFR distribution patterns. First, we assayed the morphological structure of actin in A549 cells and HeLa cells after different drug treatments to verify drug efficiency and noncell specificity (Figures 8(c)–8(j)). Subsequently, to detect the changes in the distribution patterns of EGFR on the PM under different treatment conditions, we performed three-color 3D confocal imaging on A549 cells expressing GFP-EGFR for 24 h and labelled the actin and nuclei using Alexa Fluor 647-conjugated phalloidin and DAPI, respectively. In the control group, the actin cytoskeleton was irregularly distributed throughout the cell, as clearly shown in Figure 8(c). Simultaneously, we observed that EGFR mainly existed as large clusters on the apical membrane, with a small pool of EGFR-secreting vesicles at the periphery (Figure 9(a)). According to a previous report on the GLUT4 PM cluster [23], this pattern of the EGFR PM cluster may be a consequence of fusion with retention of secretory vesicles with the PM. The peripheral secretory vesicles could be explained by the fact that the dense and disorganized actin could work as a barrier preventing them from approaching the PM. When cells were treated with 10 mM M*β*CD, actin was rearranged into orderly actin filaments compared with the untreated cells (Figure 8(d)), which was consistent with the previous statement that M*β*CD caused actin reorganization [36]. Concurrently, compared with the control group, most of the EGFR was uniformly distributed on the apical membrane with few smaller size clusters, and the peripheral EGFR secretory vesicles no longer existed (Figure 9(b)). This indicated that actin remodeling allowed EGFR secretory vesicles to undergo docking and fusion with release at the plasma membrane.

Next, we investigated whether actin barrier transient removal or reformed actin filaments play an essential role in causing uniform distribution of EGFR PM. To answer this question, A549 cells were treated with CB alone or pretreated with CB and incubated with M*β*CD. As shown in Figure 8(e), only CB treatment led to actin depolymerization into dispersed punctate structures. Under this treatment condition, we observed that a substantial level of secretory vesicles docked to the PM without fusion (Figure 9(c)). This result may be because that actin disassembly removed a cortical barrier standing in the way of secretory vesicle–PM contacts, but the disrupted actin inhibited membrane fusion, indicating the indispensable position of the complete actin structure. To further determine the role of actin in this process, the cells were incubated with CB followed by M*β*CD treatment to induce the de novo polymerization of actin into actin filaments, as demonstrated in Figure 8(f). Markedly, most of the EGFR was again uniformly distributed in smaller clusters on the apical membrane (Figure 9(d)). This observation suggested that actin depolymerization was initially required for EGFR secretory vesicles to dock with the PM, whereas the reestablishment of an actin network may act as a positive regulator to give the final stimulus to drive the final fusion with release.

These findings suggested that the dense and short actin cortex on the apical membrane may cause fusion with retention of EGFR secretory vesicles with the PM, thus contributing to the formation of larger EGFR clusters. Likewise, we believe that the nearly uniform distribution of EGFR on the basement membrane (Figure 3(b)) may be attributed to fusion with release induced by the regular arrangement of actin filaments. Altogether, these data provide new insight into the role of actin in EGFR organization, indicating a predominant influence on the distribution form of EGFR on the PM largely achieved by controlling the method of secretory vesicle–PM fusion.

## 3. Conclusion

In summary, by applying high-spatiotemporal-resolution imaging, we gained a more integrative picture of the whole process of EGFR production from synthesis to secretion onto the PM, thereby not only revealing the EGFR clustering process but also elucidating the main factors that determine the differential spatial distribution of EGFR on the PM. Our results suggest that EGFR secretory vesicles are precursors of EGFR clusters and that the secretory vesicles themselves and their methods of fusion with the plasma membrane are the main factors determining the spatial distribution of EGFR on the PM. Our work provides a deeper insight into the mechanisms underlying EGFR clustering and will therefore help to advance our understanding of EGFR cluster-related biological events, as well as lay the foundation for further characterizing the molecular mechanisms of EGFR clustering.

## 4. Materials and Methods

### 4.1. Cell Culture

A549, COS-7, and HeLa cell lines were purchased from the Shanghai Institute of Biological Sciences (Shanghai, China). All cells were cultured in Dulbecco's modified Eagle's medium (DMEM; HyClone, Thermo Scientific) supplemented with 10% fetal bovine serum (Bioind), antibiotics (Bioind), and penicillin/streptomycin (Bioind) at 37°C and 5% CO2. The cultured cells were passaged every 2 days. For confocal imaging, cells were plated in 35 mm glass-bottom petri dishes (Cellvis).

### 4.2. Cell Transfection

Cells plated on glass-bottom petri dishes and grown to 50 to 80% confluence were transiently transfected with plasmids using the transfection reagent Lipofectamine 3000 (Invitrogen) according to the manufacturer's instructions. In brief, 1 to 2 *μ*g of plasmid and 2 to 4 *μ*g of transfection reagent were mixed in Opti-MEM medium (Gibco, Thermo Fisher) and then incubated with cells. A549 cells were transiently transfected with the plasmid GFP-EGFR (Hunan Fenghui Biotechnology Co., Ltd.), GFP or EGFR-L858R mutant (provided by Prof. Hongbin Ji), and COS-7 cells were cotransfected with GFP-EGFR and DsRed-ER (Hunan Fenghui Biotechnology Co., Ltd.). Meanwhile, based on the manufacturer's instructions of Lipofectamine 3000, A549 cells were transfected with three kinds of VAMP-7 siRNAs (5′-GCGAGGAGAAAGAUUGGAAUU-3′-siRNA1; 5′-GCUCACUAUUAUCAUCAUCAU-3′-siRNA2; 5′-GCGAGUUCUCAAGUGUCUUAG-3′-siRNA3) (Sangon Biotech (Shanghai) Co., Ltd.). For siRNA rescue experiment, A549 cells were pretransfected with 1 *μ*g of VAMP-7 mt cDNA for 48 h and then transfected with VAMP-7 siRNA for another 12 h. The cells were subsequently cultured for the indicated times prior to imaging acquisition or other analysis.

### 4.3. Drug Treatment

All reagents used were acquired from Sigma–Aldrich. To block transport from the Golgi to the PM transport, cells expressing GFP-EGFR for 10 h were then incubated in fresh medium containing either 0 or 5 *μ*g/mL brefeldin A (BFA) for 20 h at 37°C. For plasma membrane cholesterol sequestration, cells were incubated in fresh medium containing 10 mM methyl-*β*-cyclodextrin (M*β*CD) for 30 min at 37°C. For actin filament cytoskeleton disruption assays, cells were incubated with 20 *μ*M cytochalasin B (CB) solutions diluted in fresh medium for 30 min at 37°C, washed three times with phosphate saline (PBS) buffer, and incubated with fresh medium or in the presence of 10 mM of M*β*CD for 30 minutes at 37°C. The above drugs were maintained in the cell culture throughout the experiments.

### 4.4. Fluorescence Labelling

For cell membrane detection, A549 cells were fixed with 4% paraformaldehyde, and then washed three times with PBS and incubated with the lipophilic dye 1,1′-dioctadecyl-3,3,3,3′-tetramethylindodicarbocyanine (DiD) (Sigma) for 30 min at room temperature. The cells were washed three times with PBS, followed by staining the cell nucleus with 20 ng/*μ*L DAPI at room temperature for 5 min.

To visualize EGFR-L858R mutant that was transiently transfected into A549 cells, the cells were first fixed with 4% paraformaldehyde (PFA) for 15 min at room temperature, followed by washing three times with PBS, then permeabilized with 0.1% TritonX-100 in PBS for 10 min at room temperature, finally blocked by 3% BSA for 30 min. After removal of the blocking solution, the cells were incubated with primary EGFR-L858R antibody (Abcam) for 60 min at room temperature. After washing the cells for three times by PBS, secondary antibody for anti-EGFR-L858R antibody (Alexa Fluor 647 goat anti-rabbit) were added into the cell culture dish for 60 min at room temperature and then rinsed with PBS.

To stain the Golgi apparatus of living cells, BODIPY TR-labelled sphingolipids (Molecular Probes) were first combined with BSA to form ceramide–BSA complexes according to the instructions. Subsequently, cells grown on glass-bottom petri dishes were rinsed with Hanks' buffered salt solution (HBSS), followed by incubation with 5 *μ*M ceramide–BSA for 30 minutes at 4°C. The cells were rinsed with ice-cold HBSS several times, and incubated in fresh medium at 37°C for an additional 30 min.

To label actin filaments, cells were rinsed with PBS, then fixed with 4% paraformaldehyde for 15 min, permeabilized and blocked with 0.1% Triton X-10 for 10 min and 2% bovine serum albumin (BSA) (Sigma) in PBS for 30 min. Afterwards, samples were rinsed twice (5 min) with PBS, and incubated with 5 *μ*g/mL Alexa Fluor 488-conjugated phalloidin or Alexa Fluor 647-conjugated phalloidin (Invitrogen) diluted in 0.2% BSA at room temperature for 1 h, and then rinsed in PBS.

### 4.5. Western Blot Analysis

Firstly, the concentration of proteins from cell lysates was determined by BCA assay kit (Pierce Chemical), then proteins were separated by 4-12% SDS-PAGE, and electrophoretically transferred to PVDF (polyvinylidene difluoride) membrane (Millipore). The membranes were blocked for 1 h in blocking buffer and incubated with antibody, mouse anti GFP monoclonal antibody (TransGen Biotech), anti pEGFR antibody (CST-2234, Cell Signaling Technologies), anti EGFR antibody (A2909, ABclonal), anti pAKT antibody (CST-4060, Cell Signaling Technologies), anti AKT antibody (CST-9272, Cell Signaling Technologies), anti pERK antibody (CST-9106, Cell Signaling Technologies), or anti ERK antibody (CST-9102, Cell Signaling Technologies) in blocking buffer at 4°C overnight. Primary antibodies were detected by incubation with HRP-conjugated secondary antibodies for 1 h at RT. Blots were developed by enhanced chemiluminescence kit (Tanon) and imaged by Tanon-5200Multi system (Tanon). Glyceraldehyde-3-phosphate dehydrogenase (GAPDH) was used as a protein-loading control.

### 4.6. Cell Growth Assay

A549 cells were seeded in triplicate in 96-well plate for 0, 24, 48, 72, and 96 h. Cells were then stained with MTT method and assessed with Epoch multivolume spectrophotometer system (570 nm/630 nm). The growth ratio was calculated by normalizing the Optical Density (OD570) obtained to the fluorescence of the first day.

### 4.7. Confocal Microscopy Imaging and Data Analysis

Confocal and time-lapse images were collected using a 100×/1.49 oil objective on a Nikon Eclipse Ti inverted microscope equipped with an Andor (Oxford Instruments) spinning disk confocal imaging system and a Yokogawa CSU-X1 and an Andor iXon Ultra electron-multiplying charge-coupled device (EMCCD) camera. The DAPI molecules used to label the cell nuclei were excited with a 405 nm laser. GFP-EGFR were excited with a 488 nm laser. DsRed-ER molecules were excited with a 561 nm laser. DiD, Alexa Fluor 647-conjugated phalloidin, and BODIPY TR-labelled sphingolipids were excited with a 640 nm laser. Live cells were imaged at 37°C, and 5% CO_2_ supplied by an incubation chamber (Tokai Hit Co., Ltd.). For time-lapse tracking, serial images could be recorded at a high frequency of several milliseconds per frame with the iXon Ultra EMCCD instrument. The microscope objective used is equipped with a piezo-actuator for fast, precise, automated closed-loop focal plane control (PRIOR). For 3D volume reconstruction, one image stack was acquired using the precise motorized stage with approximately 50-70 *z*-axis slices an axial distance of 0.2 *μ*m between two images. Then, *XY*, *XZ*, and *YZ* sections were imaged. Finally, 3D images were acquired by reconstructing maximum *z* projections of *XY* sections. The above images were acquired and processed with Andor iQ3 imaging software. In studies of differences in vesicle size at different planes of the cell, the width of vesicles was measured using ImageJ software and then analyzed in GraphPad Prism. The data shown in our work are representative of at least three independent experiments with approximately 100 cells each.

## Data Availability

The data that support the findings of this study are available within the article and its supplementary materials. Raw data are available from the corresponding authors on reasonable request.

## Supplementary Materials

Supplementary Materials Figure S1: the A549 cells were transfected with control plasmid (GFP-NC) or EGFR expression plasmid (GFP-EGFR). The expression of full GFP-EGFR was detected by western blot assay with anti-GFP antibody. Figure S2: observation of the biosynthetic trafficking route of GFP. The A549 cells alone expressing GFP for the indicated times were fixed and then imaged by confocal microscope. Scale bars = 10 *μ*m. Figure S3: visualization of the biosynthetic trafficking route of EGFR-L858R. A549 cells were transiently transfected with EGFR-L858R for indicated time and immunolabeled with anti-L858R antibody. Scale bars = 10 *μ*m. Figure S4: EGFR is localized to three-way junctions formed by interconnected ER tubules as marked by white dashed circles. The cell was cotransfected with GFP-EGFR and DsRed-ER. Scale bar = 10 *μ*m. Figure S5: effect of BFA on EGFR transport from the Golgi to the PM. The cells expressing GFP-EGFR for 10 h were chased to 30 h with or without BFA treatment at 37°C. (A) A representative confocal image of cells expressing GFP-EGFR for 10 h. (B) A representative confocal image of cells expressing GFP-EGFR for 30 h without BFA. (C) A representative confocal image of cells expressing GFP-EGFR for 10 h followed by 20 h with BFA treatment. Scale bars = 10 *μ*m. Figure S6: characterization of EGFR-L858R distribution features on the PM. A549 cells expressing EGFR-L858R for beyond 24 h. Individual cells were imaged using three-color 3D confocal microscopy. Serial confocal sections were collected from the top to the bottom of the representative cell. The dashed lines show the positions from which the *XZ* and *YZ* sections were taken. Arrowheads mark the EGFR-L858R clusters. Bar, 10 *μ*m. Figure S7: efficiency validation of VAMP-7 siRNAs knockdown. A549 cells were transfected with mock siRNA or three VAMP-7 siRNAs, respectively. Then, VAMP-7 relative mRNA levels were detected in cells under different treatment conditions. Data shown as mean ± SD. ^∗^*P* < 0.05, ^∗∗^*P* < 0.01, ^∗∗∗^*P* < 0.001; two-sided *t*-test. Figure S8: SiRNA rescue experiments. A549 cells were subjected to three different treatment conditions: transfection with mock siRNA alone, transfection with VAMP-7-1 siRNA alone, or pre-transfection with VAMP-7 mt cDNA followed by siRNA transfection. (A) Relative mRNA expression level of VAMP-7. Data shown as mean ± SD. ^∗^*P* < 0.05, ^∗∗^*P* < 0.01, ^∗∗∗^*P* < 0.001; two-sided *t*-test. (B) Western blot analysis of EGFR, p-EGFR, AKT, p-AKT, ERK, p-ERK, and GAPDH expression. (C) Relative cell growth of A549 cells. Data shown as mean ± SD. ^∗^*P* < 0.05, ^∗∗^*P* < 0.01, ^∗∗∗^*P* < 0.001; two-sided *t*-test. Movie S1: A549 cells were transfected with GFP-EGFR, and then, the dynamics of secretory vesicles on the proximal apical membrane were tracked using a real-time imaging. Movie S2: A549 cells were transfected with GFP-EGFR, and then, the dynamics of secretory vesicles on the proximal basal membrane were tracked using a real-time imaging.

## References

[1] Erich S., Sackmann E. V., Lipowsky R., Sackmann E. (1995). Biological membranes architecture and function. *Handbook of Biological Physics*.

[2] Yan Q. Y., Lu Y. T., Zhou L. L. (2018). Mechanistic insights into GLUT1 activation and clustering revealed by super-resolution imaging. *Proceedings National Academy of Sciences United States of America*.

[3] Gao J., Wang Y., Cai M. J. (2015). Mechanistic insights into EGFR membrane clustering revealed by super-resolution imaging. *Nanoscale*.

[4] Jing Y. Y., Zhou L. L., Chen J. L. (2020). Quantitatively mapping the assembly pattern of EpCAM on cell membranes with peptide probes. *Analytical Chemistry*.

[5] Chen J. L., Li H. R., Wu Q. (2021). A multidrug-resistant P-glycoprotein assembly revealed by tariquidar-probe's super-resolution imaging. *Nanoscale*.

[6] Irvine D. J., Hue K. A., Mayes A. M., Griffith L. G. (2002). Simulations of cell-surface integrin binding to nanoscale-clustered adhesion ligands. *Biophysical Journal*.

[7] Sieber J. J., Willig K. I., Heintzmann R., Hell S. W., Lang T. (2006). The SNARE motif is essential for the formation of syntaxin clusters in the plasma membrane. *Biophysical Journal*.

[8] Pontier S. M., Percherancier Y., Galandrin S., Breit A., Gales C., Bouvier M. (2008). Cholesterol-dependent separation of the *β*_2_-adrenergic receptor from its partners determines signaling efficacy:. *The Journal of Biological Chemistry*.

[9] Riese D. J., Gallo R. M., Settleman J. (2007). Mutational activation of ErbB family receptor tyrosine kinases: insights into mechanisms of signal transduction and tumorigenesis. *BioEssays*.

[10] Uribe M. L., Marrocco I., Yarden Y. (2021). EGFR in cancer: signaling mechanisms, drugs and acquired resistance. *Cancers*.

[11] Abulrob A., Lu Z., Baumann E. (2010). Nanoscale imaging of epidermal growth factor receptor clustering. *The Journal of Biological Chemistry*.

[12] Arkhipov A., Shan Y., Das R. (2013). Architecture and membrane interactions of the EGF receptor. *Cell*.

[13] Yan Q. Y., Cai M. J., Zhou L. L. (2019). Using an RNA aptamer probe for super-resolution imaging of native EGFR. *Nanoscale Advances*.

[14] Soderquist A. M., Carpenter G. (1986). Biosynthesis and metabolic degradation of receptors for epidermal growth factor. *The Journal of Membrane Biology*.

[15] Guo Y., Sirkis D. W., Schekman R. (2014). Protein sorting at thetrans-Golgi network. *Annual Review of Cell and Developmental Biology*.

[16] Bonifacino J. S., Glick B. S. (2004). The mechanisms of vesicle budding and fusion. *Cell*.

[17] Scharaw S., Iskar M., Ori A. (2016). The endosomal transcriptional regulator RNF11 integrates degradation and transport of EGFR. *The Journal of Cell Biology*.

[18] Fath S., Mancias J. D., Bi X., Goldberg J. (2007). Structure and organization of coat proteins in the COPII cage. *Cell*.

[19] Kreitzer G., Schmoranzer J., Low S. H. (2003). Three-dimensional analysis of post-Golgi carrier exocytosis in epithelial cells. *Nature Cell Biology*.

[20] Xu H. J., Wang H. D. (2021). Conventional molecular and novel structural mechanistic insights into orderly organelle interactions. *Chemical Research in Chinese Universities*.

[21] Sorkin A., Lai K. G. (2008). The reduced catalase expression in TrkA-induced cells leads to autophagic cell death via ROS accumulation. *Experimental Cell Research*.

[22] Pinilla-Macua I., Watkins S. C., Sorkin A. (2016). Endocytosis separates EGF receptors from endogenous fluorescently labeled HRas and diminishes receptor signaling to MAP kinases in endosomes. *Proceedings. National Academy of Sciences. United States of America*.

[23] Stenkula K. G., Lizunov V. A., Cushman S. W., Zimmerberg J. (2010). Insulin controls the spatial distribution of GLUT4 on the cell surface through regulation of its postfusion dispersal. *Cell Metabolism*.

[24] Wen P. J., Grenklo S., Arpino G. (2016). Actin dynamics provides membrane tension to merge fusing vesicles into the plasma membrane. *Nature Communications*.

[25] Needham S. R., Zanetti-Domingues L. C., Hirsch M. (2014). Structure–function relationships and supramolecular organization of the EGFR (epidermal growth factor receptor) on the cell surface. *Biochemical Society Transactions*.

[26] Xu H. J., Gao J., Cai M. J. (2020). Structural mechanism analysis of orderly and efficient vesicle transport by high-resolution imaging and fluorescence tracking. *Analytical Chemistry*.

[27] Chen S., Novick P., Ferro-Novick S. (2013). ER structure and function. *Current Opinion in Cell Biology*.

[28] Watanabe R., Riezman H. (2004). Differential ER exit in yeast and mammalian cells. *Current Opinion in Cell Biology*.

[29] Pagano R. E., Martin O. C., Kang H. C., Haugland R. P. (1991). A novel fluorescent ceramide analogue for studying membrane traffic in animal cells: accumulation at the Golgi apparatus results in altered spectral properties of the sphingolipid precursor. *The Journal of Cell Biology*.

[30] Miller S. G., Carnell L., Moore H. H. (1992). Post-Golgi membrane traffic: brefeldin A inhibits export from distal Golgi compartments to the cell surface but not recycling. *The Journal of Cell Biology*.

[31] Keller P., Toomre D., Díaz E., White J., Simons K. (2001). Multicolour imaging of post-Golgi sorting and trafficking in live cells. *Nature Cell Biology*.

[32] Danglot L., Chaineau M., Dahan M. (2010). Role of TI-VAMP and CD82 in EGFR cell-surface dynamics and signaling. *Journal of Cell Science*.

[33] Martinez A. S., Zahraoui A., Daniel L., Thierry G. (2000). Role of tetanus neurotoxin insensitive vesicle-associated membrane protein (TI-VAMP) in vesicular transport mediating neurite outgrowth. *The Journal of Cell Biology*.

[34] Fujita M., Kinoshita T. (2012). GPI-anchor remodeling: potential functions of GPI-anchors in intracellular trafficking and membrane dynamics. *Biochimica et Biophysica Acta-Molecular and Cell Biology of Lipids*.

[35] Ruan H., Yu J., Yuan J., Li N., Fang X. (2016). Nanoscale distribution of transforming growth factor receptor on post-Golgi vesicle revealed by super-resolution microscopy. *Chemistry, an Asian Journal*.

[36] de Oliveira Andrade L. (2016). Understanding the role of cholesterol in cellular biomechanic and regulation of vesicular trafficking: the power of imaging. *Biomed Spectrosc Imaging*.

