# Supplementary figures and images for: Mechanistic Insights into Membrane Protein Clustering Revealed by Visualizing EGFR Secretion
Source: Research (Wash D C). 2022 Oct 16;2022:9835035. doi: 10.34133/2022/9835035 (PMC9620640; doi:10.34133/2022/9835035)

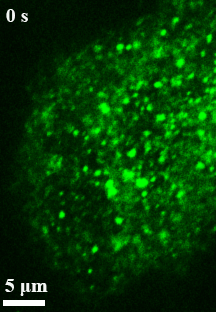

Supplement: Supplementary Materials — Figure S1: the A549 cells were transfected with control plasmid (GFP-NC) or EGFR expression plasmid (GFP-EGFR). The expression of full GFP-EGFR was detected by western blot assay with anti-GFP antibody. Figure S2: observation of the biosynthetic trafficking route of GFP. The A549 cells alone expressing GFP for the indicated times were fixed and then imaged by confocal microscope. Scale bars = 10 μm. Figure S3: visualization of the biosynthetic trafficking route of EGFR-L858R. A549 cells were transiently transfected with EGFR-L858R for indicated time and immunolabeled with anti-L858R antibody. Scale bars = 10 μm. Figure S4: EGFR is localized to three-way junctions formed by interconnected ER tubules as marked by white dashed circles. The cell was cotransfected with GFP-EGFR and DsRed-ER. Scale bar = 10 μm. Figure S5: effect of BFA on EGFR transport from the Golgi to the PM. The cells expressing GFP-EGFR for 10 h were chased to 30 h with or without BFA treatment at 37°C. (A) A representative confocal image of cells expressing GFP-EGFR for 10 h. (B) A representative confocal image of cells expressing GFP-EGFR for 30 h without BFA. (C) A representative confocal image of cells expressing GFP-EGFR for 10 h followed by 20 h with BFA treatment. Scale bars = 10 μm. Figure S6: characterization of EGFR-L858R distribution features on the PM. A549 cells expressing EGFR-L858R for beyond 24 h. Individual cells were imaged using three-color 3D confocal microscopy. Serial confocal sections were collected from the top to the bottom of the representative cell. The dashed lines show the positions from which the XZ and YZ sections were taken. Arrowheads mark the EGFR-L858R clusters. Bar, 10 μm. Figure S7: efficiency validation of VAMP-7 siRNAs knockdown. A549 cells were transfected with mock siRNA or three VAMP-7 siRNAs, respectively. Then, VAMP-7 relative mRNA levels were detected in cells under different treatment conditions. Data shown as mean ± SD. ∗P < 0.05, ∗∗P < 0.01, ∗∗∗P < 0.00 [file 9835035.f1.zip › Movie S1.gif]

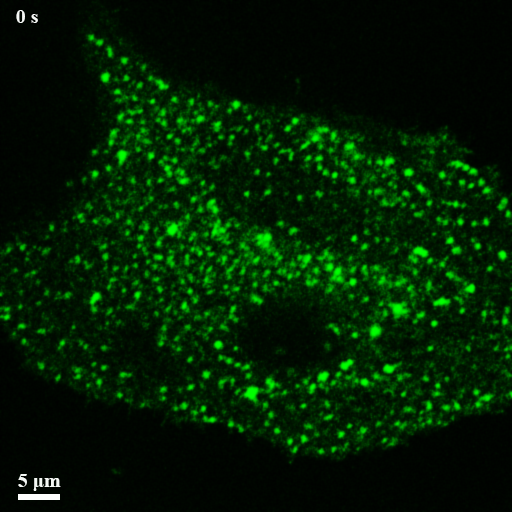

Supplement: Supplementary Materials — Figure S1: the A549 cells were transfected with control plasmid (GFP-NC) or EGFR expression plasmid (GFP-EGFR). The expression of full GFP-EGFR was detected by western blot assay with anti-GFP antibody. Figure S2: observation of the biosynthetic trafficking route of GFP. The A549 cells alone expressing GFP for the indicated times were fixed and then imaged by confocal microscope. Scale bars = 10 μm. Figure S3: visualization of the biosynthetic trafficking route of EGFR-L858R. A549 cells were transiently transfected with EGFR-L858R for indicated time and immunolabeled with anti-L858R antibody. Scale bars = 10 μm. Figure S4: EGFR is localized to three-way junctions formed by interconnected ER tubules as marked by white dashed circles. The cell was cotransfected with GFP-EGFR and DsRed-ER. Scale bar = 10 μm. Figure S5: effect of BFA on EGFR transport from the Golgi to the PM. The cells expressing GFP-EGFR for 10 h were chased to 30 h with or without BFA treatment at 37°C. (A) A representative confocal image of cells expressing GFP-EGFR for 10 h. (B) A representative confocal image of cells expressing GFP-EGFR for 30 h without BFA. (C) A representative confocal image of cells expressing GFP-EGFR for 10 h followed by 20 h with BFA treatment. Scale bars = 10 μm. Figure S6: characterization of EGFR-L858R distribution features on the PM. A549 cells expressing EGFR-L858R for beyond 24 h. Individual cells were imaged using three-color 3D confocal microscopy. Serial confocal sections were collected from the top to the bottom of the representative cell. The dashed lines show the positions from which the XZ and YZ sections were taken. Arrowheads mark the EGFR-L858R clusters. Bar, 10 μm. Figure S7: efficiency validation of VAMP-7 siRNAs knockdown. A549 cells were transfected with mock siRNA or three VAMP-7 siRNAs, respectively. Then, VAMP-7 relative mRNA levels were detected in cells under different treatment conditions. Data shown as mean ± SD. ∗P < 0.05, ∗∗P < 0.01, ∗∗∗P < 0.00 [file 9835035.f1.zip › Movie S2.gif]
